# Pulmonary Microbial Composition in Sepsis-Induced Acute Respiratory Distress Syndrome

**DOI:** 10.3389/fmolb.2022.862570

**Published:** 2022-06-23

**Authors:** Peng Zhang, Baoyi Liu, Weihao Zheng, Yantang Chen, Zhentao Wu, Yuting Lu, Jie Ma, Wenjie Lu, Mingzhu Zheng, Wanting Wu, Zijie Meng, Jinhua Wu, Yan Zheng, Xin Zhang, Shuang Zhang, Yanming Huang

**Affiliations:** ^1^ Department of Critical Care Medicine, Jiangmen Central Hospital, Jiangmen, China; ^2^ Clinical Experimental Center, Jiangmen Key Laboratory of Clinical Biobanks and Translational Research, Jiangmen Central Hospital, Jiangmen, China; ^3^ Department of Clinical Laboratory, Jiangmen Central Hospital, Jiangmen, China; ^4^ Department of Research and Development, Guangdong Research Institute of Genetic Diagnostic and Engineering Technologies for Thalassemia, Hybribio Limited, Guangzhou, China; ^5^ Dongguan Key Laboratory of Medical Bioactive Molecular Developmental and Translational Research, Guangdong Provincial Key Laboratory of Medical Molecular Diagnostics, Guangdong Medical University, Dongguan, China; ^6^ Collaborative Innovation Center for Antitumor Active Substance Research and Development, Guangdong Medical University, Zhanjiang, China; ^7^ Department of Respiratory and Critical Care Medicine, Jiangmen Central Hospital, Jiangmen, China

**Keywords:** acute respiratory distress syndrome(ARDS), sepsis, metagenomic next-generation sequencing(mNGS), pulmonary microbiome, acute respiratory distress syndrome of pulmonary, acute respiratory distress syndrome of extrapulmonary

## Abstract

**Background:** Acute respiratory distress syndrome (ARDS) is an unresolved challenge in the field of respiratory and critical care, and the changes in the lung microbiome during the development of ARDS and their clinical diagnostic value remain unclear. This study aimed to explore the role of the lung microbiome in disease progression in patients with sepsis-induced ARDS and potential therapeutic targets.

**Methods:** Patients with ARDS were divided into two groups according to the initial site of infection, intrapulmonary infection (ARDSp, 111 cases) and extrapulmonary infection (ARDSexp, 45 cases), and a total of 28 patients with mild pulmonary infections were enrolled as the control group. In this study, we sequenced the DNA in the bronchoalveolar lavage fluid collected from patients using metagenomic next-generation sequencing (mNGS) to analyze the changes in the lung microbiome in patients with different infectious site and prognosis and before and after antibiotic treatment.

**Results:** The Shannon–Wiener index indicated a statistically significant reduction in microbial diversity in the ARDSp group compared with the ARDSexp and control groups. The ARDSp group was characterized by a reduction in microbiome diversity, mainly in the normal microbes of the lung, whereas the ARDSexp group was characterized by an increase in microbiome diversity, mainly in conditionally pathogenic bacteria and intestinal microbes. Further analysis showed that an increase in *Bilophila* is a potential risk factor for death in ARDSexp. An increase in *Escherichia coli*, *Staphylococcus aureus*, *Candida albicans*, enteric microbes, or conditional pathogens may be risk factors for death in ARDSp. In contrast, *Hydrobacter* may be a protective factor in ARDSp.

**Conclusion:** Different initial sites of infection and prognoses are likely to affect the composition and diversity of the pulmonary microbiome in patients with septic ARDS. This study provides insights into disease development and exploration of potential therapeutic targets.

## 1 Introduction

Acute respiratory distress syndrome (ARDS) is a clinical syndrome that occurs during severe infections, shock, trauma, and burns and is mostly caused by sepsis (Force et al., 2012; Matthay et al., 2019). It is characterized by hypoxemia and respiratory distress. Owing to its rapid progression, high mortality rate, and lack of effective treatments, ARDS is one of the leading causes of death in patients with acute and critical illnesses. In ARDS, damage to alveolar epithelial cells and pulmonary capillary endothelial cells causes increased alveolar-capillary permeability and the collection of protein-laden edema fluid in the alveolar lumen, eventually leading to diffuse interstitial lung edema (Matthay et al., 2019). In recent years, there has been increasing interest in research on ARDS, and the exploration of timely and effective treatments and prognostic markers for ARDS remains a hot topic of research.

In a previous study, we found that the use of metagenomic next-generation sequencing (mNGS) for ARDS caused by severe pneumonia improved clinical diagnosis and guided the clinical use of drugs, thereby improving patient prognosis (Zhang et al., 2020). In addition, changes in the lung microbiome in patients can be explored using mNGS. Research on the relationship between the lung microenvironment and the etiology of ARDS is still in its early stages. Dickson et al. (2018) suggested the presence of interactions between alterations in the pulmonary microbiome and ARDS. ARDS infections or other lung injuries can directly alter the lung microbiome, including ventilator-induced injury and aspiration. Alterations in the pulmonary microbiome may, in turn, contribute to lung injury by promoting increased pulmonary vascular permeability, the establishment of stark oxygen gradients, a surge in inflammatory molecules for bacterial growth (Freestone et al., 2012; Dickson et al., 2015), and damage to host defenses, ultimately altering the alveolar microenvironment. Once both lung microbial imbalance and lung injury occur, they interact via a positive feedback loop. Therefore, regulation of the microbiome is likely to be a potential therapeutic or prophylactic target for ARDS (Dickson, 2016). However, the relationship between alterations in the microbiome and ARDS and their influence on disease regression and prognosis remain to be further evaluated.

This study aimed to explore the role of the lung microbiome in disease progression in patients with sepsis-induced ARDS. Moreover, potential therapeutic targets were screened based on changes in the microbiome in the lung microenvironment.

## 2 Materials and Methods

### 2.1 Ethics and Informed Consent

The study protocol was reviewed and approved by the Ethics Review Committee of Jiangmen Central Hospital (No. 2019-15). Written informed consent was obtained from the patients or their legal representatives before the collection of bronchoalveolar lavage fluid (BALF) samples by bronchoalveolar lavage using fiberoptic bronchoscopy.

### 2.2 Patients

A retrospective analysis was conducted on patients with ARDS caused by sepsis who were admitted to the intensive care unit (ICU) of Jiangmen Central Hospital from January 2018 to June 2021. The inclusion criteria were as follows: 1) the diagnosis of ARDS met the Berlin 2012 definition (Force et al., 2012), 2) the etiology of ARDS was sepsis, 3) the age was greater than 18 years, and 4) the clinical profile was complete. The exclusion criteria were as follows: 1) ARDS caused by non-infectious factors, 2) age <18 years, and 3) an incomplete clinical profile. The patients were divided into two groups according to their initial infection status: intrapulmonary infection-induced ARDS (ARDSp group) and extrapulmonary infection-induced ARDS (ARDSexp group). The ARDSp group included 111 patients, the ARDSexp group included 45 patients, and the control group included 28 patients, including patients with mild pulmonary infection and non-ARDS, all of whom had a good prognosis and did not return to the ICU within 90 days of being transferred out of the ICU.

### 2.3 General Treatment Plan

All patients with sepsis were treated according to the sepsis guidelines (Rhodes et al., 2017) and empirical anti-infective therapy in conjunction with clinical indicators of infection and imaging information. Patients with ARDS were mechanically ventilated according to the ARDS ventilation guidelines (Bein et al., 2016; Griffiths et al., 2019), and the anti-infective regimen was adjusted according to the patient’s inflammatory indicators, imaging data, and microbiological tests.

### 2.4 BALF Collection Process

All patients were intubated and mechanically ventilated in the ICU, and BALF specimens were obtained using a fiberoptic bronchoscope (Chinese Thoracic Society, 2017). Baseline specimens were collected within 24 h of ARDS diagnosis in the ICU before antibiotic administration. Some patients were treated for 7 days, and post-treatment specimens were retained. The baseline and post-treatment specimens were sent to the laboratory for pathogenic culture. The remaining specimens were also sent to the clinical experimental center for DNA extraction and stored at -20°C for research purposes. All laboratory consumables were purchased from Guangzhou Jet Bio-Filtration Co., Ltd. (China). The final specimens were sent to Guangdong Longsee Biomedical Co., Ltd. for metagenomic sequencing, including Bacterial nucleic acid purification, DNA library preparation, high-throughput sequencing, bioinformatics analysis, and pathogenic data interpretation (Miao et al., 2018).

### 2.5 Experimental Groups

**FIGURE 1:**
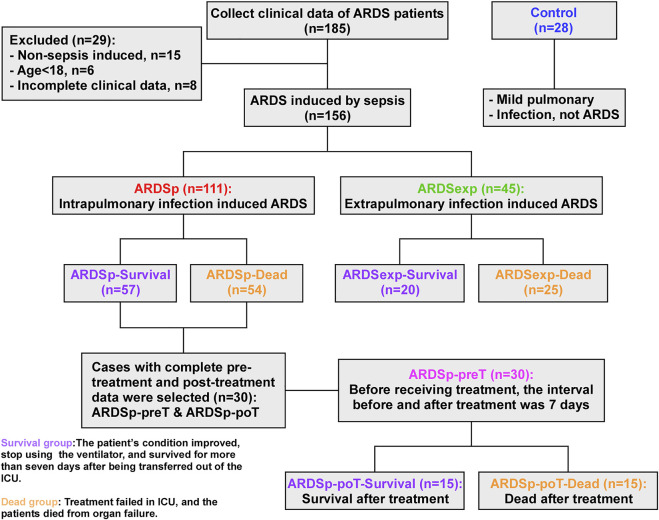
Schematic diagram of the experimental groupings.

As shown in Figure 1, patients in all groups were stratified according to their prognosis. Those who improved in the ICU with treatment and ventilation and could be successfully transferred out of the ICU within 7 days were defined as the “survival group,” while those who did not benefit from ICU care and died of organ failure were defined as the “dead group.” Based on different etiologies with different prognoses, 57 cases were classified as the ARDSp-survival group, 54 cases as the ARDSp-dead group, 20 cases as the ARDSexp-survival group, and 25 cases as the ARDSexp-dead group. We analyzed the similarities and differences in the microbiome between the different groups and searched for microbial markers associated with prognosis using metagenomic DNA sequencing of the BALF collected from these patients.

Thirty patients with complete data in the ARDSp group, including pre-treatment and post-treatment data, were selected and divided into the pre-treatment group (ARDSp-preT group) and post-treatment group (ARDSp-poT group). The ARDSp-poT-survival (post-treatment) group was defined as the post-treatment-survival group, with 15 cases, based on the improvement after ICU care allowing the cessation of ventilator use and survival within 7 days of transfer out of ICU transfer. Conversely, the ARDSp-poT-dead group was defined as a failure of treatment in the ICU and death due to organ failure (15 cases). The control group included 28 cases. The similarities and differences in the microbiome between the different groups were also analyzed.

### 2.6 Pathogenic and Background Microorganisms

In total, 2,728 microorganisms were sequenced in this study. The MetaPhlAn database was used for the taxonomic assignment (Truong et al., 2015). As the RPM values of different microorganisms varied considerably, the microorganisms were divided into pathogenic and background microorganisms and were analyzed separately. Pathogenic microorganisms had significantly higher RPM values than the background microorganisms. We extracted common nosocomial infection pathogenic microorganisms, including common bacteria, fungi, viruses, and specific pathogens, based on 2019 CHINET surveillance data (Hu et al., 2020) and common pathogenic microorganisms in sequencing laboratory testing, and defined 57 microorganisms as pathogenic microorganisms (Supplementary Table S1). The remaining microorganisms were considered background microorganisms, with a total of 1,040 at the genus level.

### 2.7 Metagenomic Next-Generation Sequencing and Analysis

#### 2.7.1 Nucleic Acid Extraction and Library Preparation

DNA was extracted using nucleic acid extraction kit (#20150013, Hybribio, China) and stored at -20°C to prevent degradation.

A DNA library was constructed using the VAHTS® Universal Plus DNA Library Prep Kit for Illumina (Guangdong Longsee Biomedical Co., Ltd, China). All experiments were performed on ice. The reaction system was prepared in sterile PCR tubes according to the manufacturer’s instructions, and DNA fragmentation and adapter ligation were performed by polymerase chain reaction (PCR). The products were purified using VAHTS DNA Clean Beads. Library amplification was then performed by PCR and the amplified products were purified again. PCR primer information is shown in Supplementary Table S2.

#### 2.7.2 Bioinformatics Analysis

After library preparation, high-throughput sequencing was performed using the NextSeq CN500 sequencing platform, and sequencing data were formatted using the official Illumina software bcl2fastq to obtain FASTQ files for individual samples. Quality control was performed using the quality control software fastp (version 0.20.0) to remove bases with an average qc value <20 within 4 bp of the end and to remove reads <70 bp in length in the FASTQ files. After quality control, the host genome was removed by matching the quality-controlled reads to the human genome (hg39) using Bowtie (version 1.2.2) and retaining unmatched reads for subsequent analysis. After removing the human reads, the remaining reads were matched to a reference genome database of five pathogenic microorganisms (Longsee Clinical Pathogen Sequence Database) using bowtie2 (version 2.3.4.1) in an end-to-end matching mode. The results were further standardized in Python and R before analysis (RPM = number of reads on match/total number of reads ∗ 1000000).

### 2.8 Statistical Analysis

We divided the microorganisms into pathogenic and background microorganisms, and counted the number of positive and negative sequencing results for each group separately. Among pathogenic microorganisms, those with RPM values ≥1 were defined as positive and those with RPM values <1 as negative; among background microorganisms, those with RPM values >0 were defined as positive and those with RPM values = 0 as negative.

To analyze the positive rate of the microbiota, we used the chi-square test or Fisher’s test and presented the data using a histogram. To analyze the abundance of the microbiota, we log2-processed the RPM values from the sequencing results, compared the median values using a paired t-test, and presented the data using a heatmap. Analyses were performed using GraphPad 9.3 or R4.1.2. Statistical significance was set as *p* < 0.05. Cox and cluster analyses were performed using the SPSS software (version 26.0) and multiple experiment viewer, respectively. Principal component analysis (PCA) was performed using GraphPad prism 9.3 and the Adonis analysis was performed on the Omicshare platform. The Shannon–Wiener index was calculated as follows: H=∑(pi)(ln⁡pi).

## 3 Results

### 3.1 Patient Clinical Characteristics

A total of 156 patients with sepsis-induced ARDS were selected for this study and were divided into two groups according to their initial infection status: intrapulmonary infection-induced ARDS (ARDSp, n = 111), and extrapulmonary infection-induced ARDS groups (ARDSexp, n = 45), and a control group (n = 28, Figure 1). No significant statistical difference in age and sex was found between the ARDS and control groups (Table 1). The ARDS group was divided into the ARDSp group and the ARDSexp group for comparison, but there was no significant statistical difference in the basic characteristics of the two groups. (Table 2). Pre-treatment laboratory tests showed that the ARDSexp group had significantly different results compared with the ARDSp group, including PCT (*p* < 0.001), PLT (*p* = 0.011), Scr (*p* = 0.041), T. Bil (*p* = 0.014), Lac (*p* < 0.001), pre-treatment APACHE II (*p* < 0.001), and the SOFA score (*p* < 0.001; Table 3). In addition, a higher number of patients in the ARDSexp group required treatment with vasoactive drugs (*p* < 0.001) and CRRT (*p* < 0.001) than in the ARDSp group, with no statistical difference in the 90-day all-cause mortality (Table 4). These findings suggest that patients in the ARDSexp group were sicker than those in the ARDSp group; however, there was no significant difference in the prognosis between the two groups.

**TABLE 1:** General information for the ARDS group versus the control group.

	ARDS (n = 156)	Control (*n* = 28)	*p* value
Age (years)			
≥60, n (%)	95 (60.9)	13 (46.4)	0.152
<60, n (%)	61 (39.1)	15 (53.6)	
Gender			
Male, n (%)	106 (67.9)	16 (57.1)	0.265
Female, n (%)	50 (32.1)	12 (42.9)	

**TABLE 2:** Patient characteristics and baseline information for the ARDS group versus the ARDSexp group.

	ARDSp (*n* = 111)	ARDSexp (*n* = 45)	*p* value
Age (years)			
≥60, n (%)	68 (61.3)	27 (60.0)	0.884
<60, n (%)	43 (38.7)	18 (40.0)	
Gender			
Male, n (%)	77 (69.4)	29 (64.4)	0.550
Female, n (%)	34 (30.6)	16 (35.6)	
Underlying diseases			
Hypertension, n (%)	35 (31.5)	11 (24.4)	0.379
Coronary heart disease, n (%)	11 (9.9)	3 (6.7)	0.521
COPD, n (%)	19 (17.1)	4 (8.9)	0.189
Chronic nephrosis, n (%)	12 (10.8)	3 (6.7)	0.426
Diabetes, n (%)	19 (17.1)	11 (24.4)	0.293
Immunosuppression, n (%)	15 (13.5)	3 (6.7)	0.225
Tumor, n (%)	9 (8.1)	4 (8.9)	0.873
Smoking, n (%)	35 (31.5)	8 (17.8)	0.082
Drinking, n (%)	14 (12.6)	4 (8.9)	0.435
Primary site of infection			
Lung, n (%)	111 (100)	0 (0)	—
Blood, n (%)	0 (0)	7 (15.6)	—
Gastrointestinal tract, n (%)	0 (0)	18 (40.0)	—
Liver, gallbladder, and pancreas, n (%)	0 (0)	9 (20.0)	—
Skin, n (%)	0 (0)	10 (22.2)	—
Urinary system, n (%)	0 (0)	1 (2.2)	

The difference between the two groups was tested by the chi-square test. *p* < 0.05 was considered statistically significant. COPD, chronic obstructive pulmonary disease.

**TABLE 3:** Comparison of laboratory examination, ventilator parameters, APACHE II score, and SOFA score before treatment between the two groups of patients.

	ARDSp (*n* = 111)	ARDSexp (*n* = 45)	*p* value
Laboratory examination before treatment			
PCT (ug/L)	1.9 (0.4, 10.0)	16.2 (6.0, 72.8)	< 0.001*
WBC (109/L)	13.0 (6.9, 17.2)	13.2 (8.2, 19.2)	0.197
PLT (109/L)	176 (112, 218)	115 (57, 204)	0.011*
Scr (μmol/L)	90 (70, 188)	142 (89, 260)	0.041*
T.Bil (mmol/L)	12.4 (7.4, 22.9)	21.0 (11.3, 50.9)	0.014*
ALT (IU/L)	24 (13, 51)	37 (25, 87)	0.259
Lac (mmol/L)	1.8 (1.2, 2.8)	3.6 (1.7, 7.6)	< 0.001*
OI	148 (106, 181)	164 (140, 210)	0.084
APACHE II score before treatment	21 (18, 23)	22 (19, 25)	< 0.001*
SOFA score before treatment	7 (5, 8)	8 (6, 10)	< 0.001*

Patient physiological index measurements are presented as median (interquartile). *p* < 0.05 is considered statistically significant. PCT, procalcitonin; WBC, white blood cell; PLT, platelet; Scr, serum creatinine; T.Bil, total bilirubin; ALT, alanine aminotransferase; Lac, lactic acid; OI, oxygen index; APACHE, acute physiology and chronic health evaluation; SOFA, sequential organ failure assessment.

**TABLE 4:** Comparison of special ICU treatment and prognosis between two groups of patients.

	ARDSp (*n* = 111)	ARDSexp (*n* = 45)	*p* value
Use of vasoactive drugs, n (%)	51 (45.9)	39 (86.7)	< 0.001*
CRRT, n (%)	13 (11.7)	20 (44.4)	< 0.001*
ECMO, n (%)	8 (7.2)	0 (0)	0.106
Prone ventilation, n (%)	22 (19.8)	3 (6.7)	0.053
All-cause mortality at 90 days, n (%)	54 (48.6)	25 (55.6)	0.619

The difference between the two groups was tested by chi-square test. *p* < 0.05 was considered statistically significant. CRRT, continuous renal replacement therapy; ECMO, extracorporeal membrane oxygenation.

### 3.2 Comparison of the Lung Microbiome Between the ARDSp and ARDSexp Groups

**FIGURE 2:**
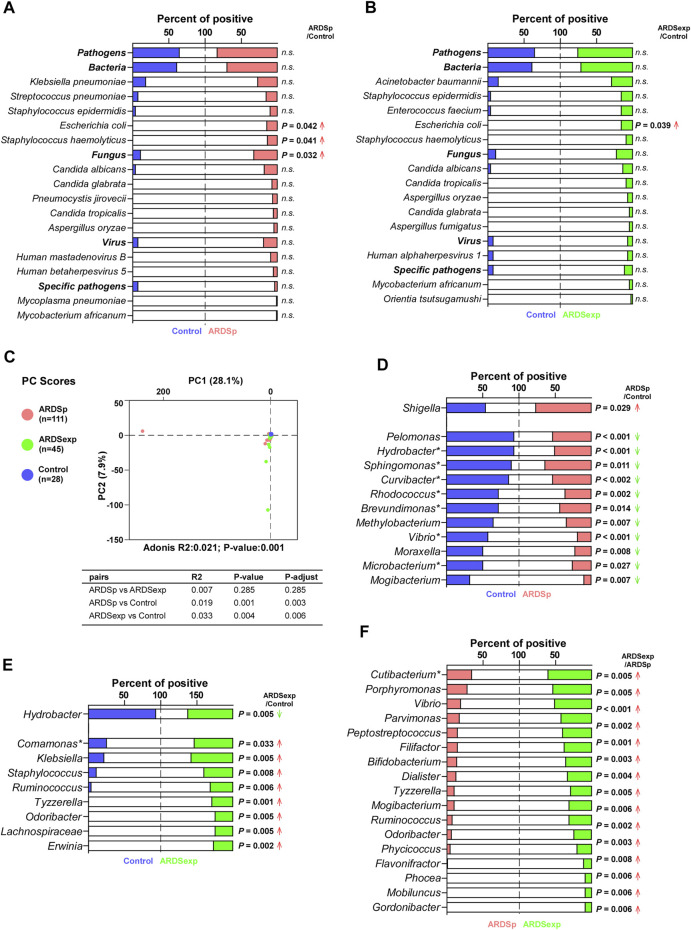
Comparison of the pulmonary microbiome between the ARDSp and ARDSexp groups. Comparison of pathogenic microorganisms. **(A)** Compared with the control group, the ARDSp group had a higher positive rate for *Escherichia coli*, *Staphylococcus haemolyticus,* and *Fungus.* **(B)** Compared with the control group, the ARDSexp group had a higher positive rate for *Escherichia coli*. Comparison of background microorganisms: **(C)** PCA showed that the community composition of ARDSp and ARDSexp groups was different from that of the control group. **(D)** ARDSp group had a predominantly reduced positive rate for the ARDSp group compared with the control group, except for *Shigella*. **(E)** ARDSexp group showed a predominant increase in the positive rate in the ARDSexp group compared with the control group, except for *Hydrobacter*. **(F)** ARDSexp group has a higher positive rate of the microbiome compared with the ARDSp group. R2: variation; P-adjust: *p* value was adjusted by using the Benjamini–Hochberg (BH) method. Note: asterisk represents the microbiome with a simultaneous increase or decrease in positive rate and abundance comparisons.

ARDS may be caused by several pathogenic microorganisms, including bacteria, fungi, viruses, and specific pathogens. We first compared the composition and abundance of pathogenic microorganisms among the control, ARDSp, and ARDSexp groups. Shannon’s diversity index results suggested that the ARDSp group had less microbiome diversity compared with the ARDSexp and control groups (Supplementary Figure 7A). Further analysis revealed that the ARDSp group had a higher positive rate for *Escherichia coli*, *Staphylococcus haemolyticus*, and fungi than the control group (Figure 2A); the ARDSexp group had a higher positive rate for *Escherichia coli* than both groups (Figure 2B). No statistically significant difference in the pathogenic microorganisms was found between the ARDSp and ARDSexp groups (Supplementary Figure 1A).

Furthermore, we performed PCA on background microorganisms among the control, ARDSp, and ARDSexp groups. As shown in the graph (Figure 2C), the control group had the most concentrated community composition, whereas both the ARDSp and ARDSexp groups had a larger number of different microorganism species than the control group. This indicates that the community composition of the background microorganisms was relatively consistent in the control group, and there were a few abnormally increased background microorganisms in the ARDSp and ARDSexp groups. In a subsequent comparison between the ARDSp, ARDSexp, and control groups, we found that the ARDSp group was characterized by a reduction in both positivity and abundance compared with the control group (Figure 2D; Supplementary Figure 1B), suggesting that the ARDSp microbiome was characterized by a reduction in diversity. Therefore, we speculate that an increase in pathogenic microorganisms inhibits the growth of the “normal” respiratory microbiome. Interestingly, increased positivity and an abundance of intestinal microbes such as *Shigella* in ARDSp were observed. In contrast, the ARDSexp group presented a predominant increase in the positivity of microbes including *Comamonas*, *Klebsiella*, *Staphylococcus*, *Ruminococcus*, *Tyzzerella*, *Odoribacter*, Lachnospiraceae, and *Erwinia,* implying that the ARDSexp microbiome was characterized by an increased diversity of the microbiome (Figure 2E; Supplementary Figure 1C), most of which are of intestinal origin. In addition, the ARDSexp group had a higher positive rate and abundance of microbes than the ARDSp group such as *Cutibacterium* (Figure 2F; Supplementary Figure 1D), which were also mainly of intestinal origin.

### 3.3 Microbial Analysis Associated With the Prognosis of the ARDSp Group

**FIGURE 3:**
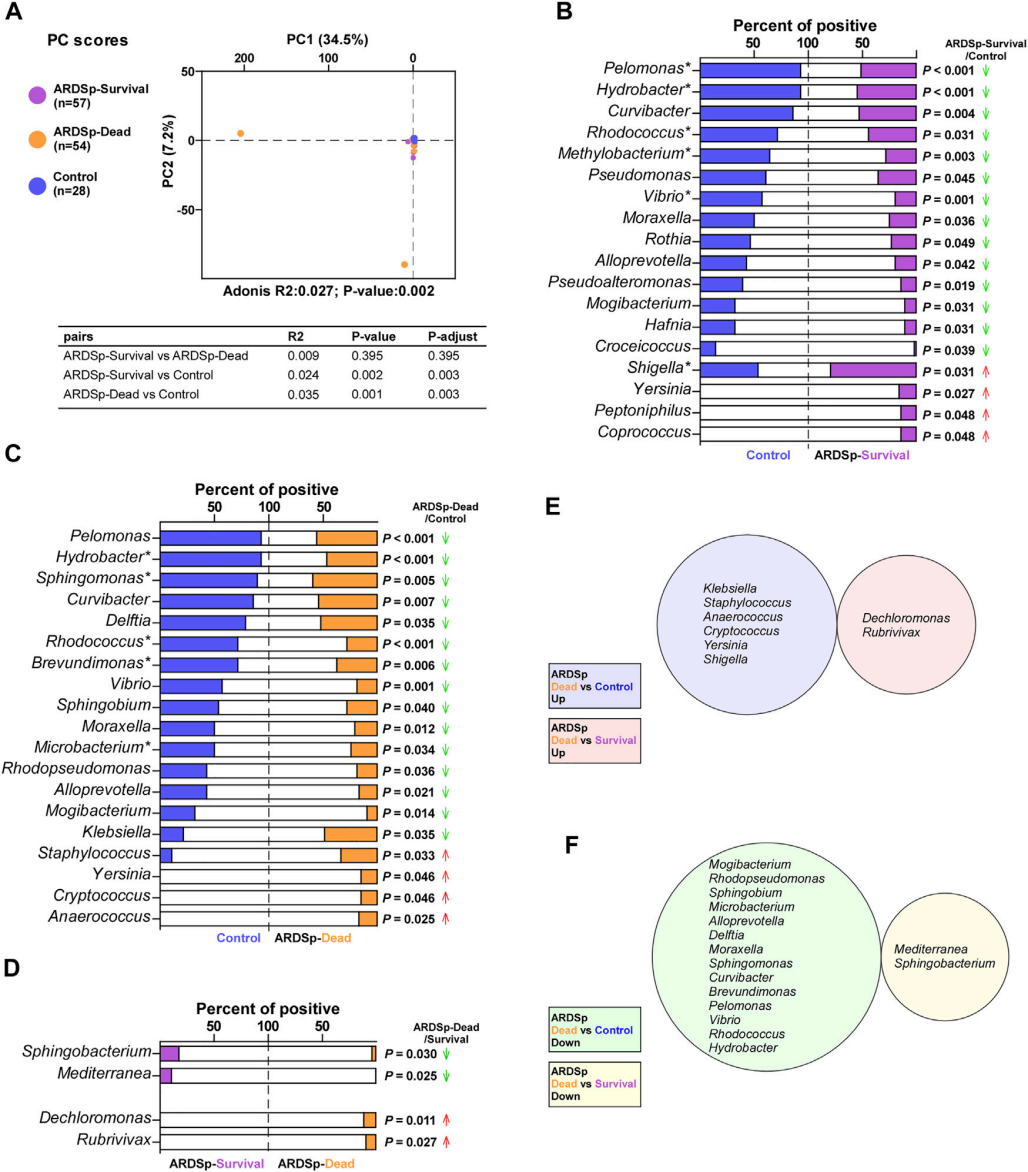
Microbial analysis associated with the prognosis of the ARDSp group. Comparison of background microorganisms: **(A)** PCA showed the community composition of ARDSp-survival and ARDSp-dead groups was significantly different from that of the control group. **(B)** ARDSp-survival group had a predominantly reduced rate of positivity compared with the control group. **(C)** ARDSp-dead group also had a predominantly reduced positive rate when compared with the control group. **(D)** There were four pathogenic microorganisms with statistically significant differences in the positive rate in the ARDSp-dead group compared with the ARDSp-survival group. **(E)** No simultaneous increases in the background microbiome were found in the ARDSp-dead group compared with the control and ARDSp-survival groups. **(F)** No simultaneous decreases in the background microbiome were found in the ARDSp-dead group compared with the control and ARDSp-survival groups. R2: variation; P-adjust: *p* value was adjusted by using the Benjamini–Hochberg (BH) method.

We divided the ARDSp group into ARDSp-survival and ARDSp-dead groups according to different prognoses and performed PCA together with the control group (Figure 3A). PCA results showed that there was a relatively large amount of taxonomic overlap between the control and ARDSp-survival groups, with more obviously isolated specimens in the ARDSp-dead group. This suggests that the control and ARDSp-survival groups had a relatively similar microbial community composition, whereas the ARDSp-dead group had several abnormally increased background microorganisms.

The pathogenic microorganisms and background microbiome that increased or decreased simultaneously in the ARDSp-dead group compared with the control and ARDSp-survival groups are probably related to prognosis. Therefore, we compared the composition and abundance of microorganisms among these three groups. The Shannon index result suggested that the ARDSp group had less diversity than the control group; however, there was no statistically significant difference in the microbial diversity between the ARDSp-survival and ARDSp-dead groups (Supplementary Figure 2B). In addition, the ARDSp-dead group had a significantly higher positive rate for pathogenic microorganisms including total pathogens, total fungi, *Staphylococcus haemolyticus,* and *Escherichia coli* than the control group (Supplementary Figure 2B). Compared with the ARDSp-survival group, the ARDSp-dead group had a significantly higher positive rate for *Pseudomonas aeruginosa* (Supplementary Figure 2C).

Moreover, the ARDSp-survival and ARDSp-dead groups showed a predominant decrease in the positivity and abundance of background microorganisms compared with the control group (Figures 3B–D; Supplementary Figure 2E,F). Nevertheless, no prognosis-related microbes were identified among pathogenic or background microorganisms (Figures 3E,F).

### 3.4 Microbial Analysis Associated With the Prognosis of the ARDSexp Group

**FIGURE 4:**
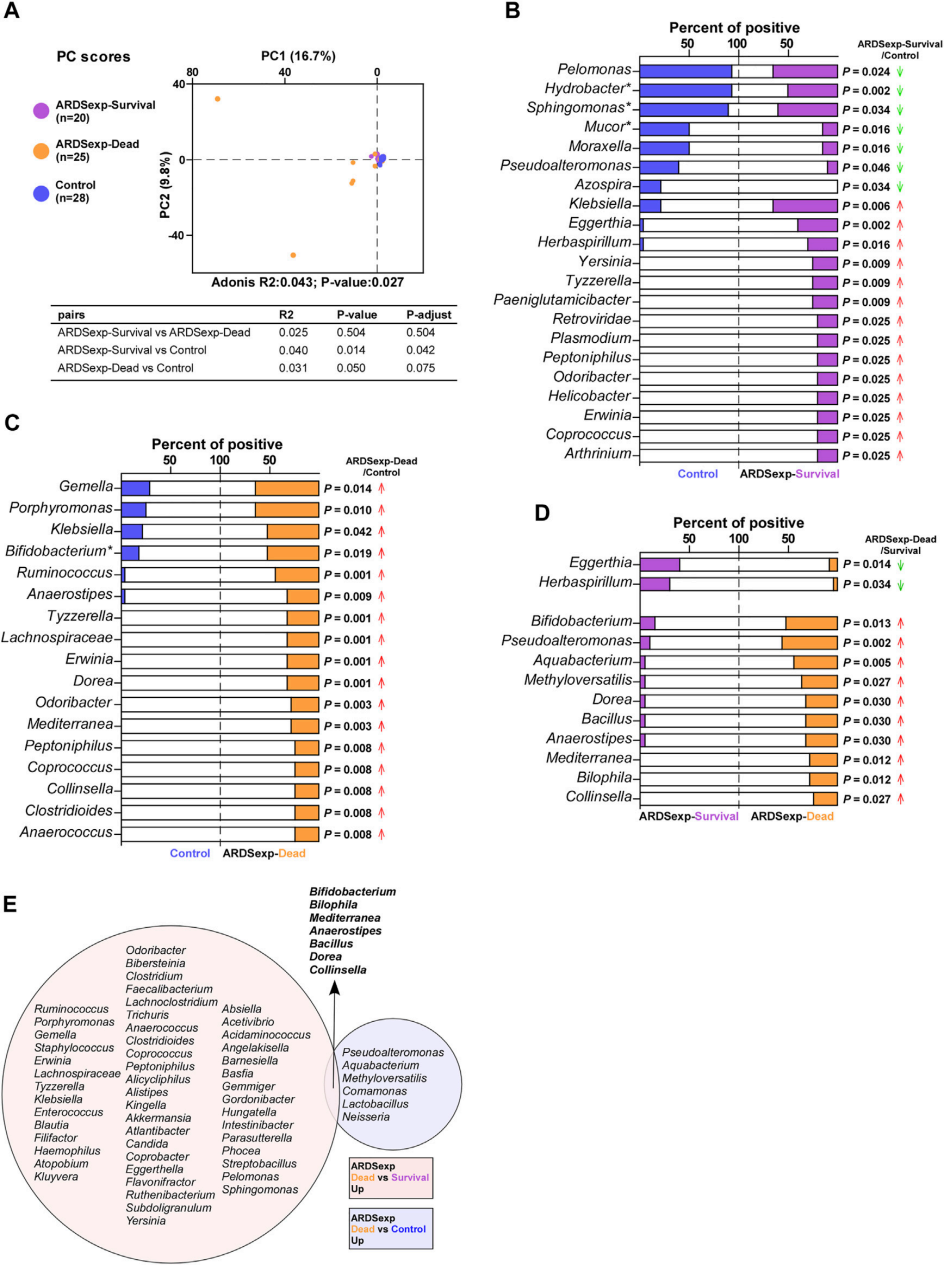
Microbial analysis associated with the prognosis of the ARDSexp group. Comparison of background microorganisms: **(A)** PCA showed that the community composition of the ARDSexp-dead group was different from that of the control group. **(B)** ARDSexp-survival group had a predominantly elevated positive rate compared with the control group. **(C)** ARDSexp-dead group had an elevated positive rate compared with the control group. **(D)** ARDSexp-dead group had a predominantly elevated positive rate when compared with the ARDSexp-survival group. **(E)** ARDSexp-dead group had seven increased background microbiomes, *Bifidobacterium*, *Bilophila*, *Mediterranea*, *Anaerostipes*, *Bacillus*, *Dorea*, and *Collinsella* compared with the control and ARDSexp-survival groups. R2: variation; P-adjust: *p* value was adjusted by using the Benjamini–Hochberg (BH) method.

We divided the ARDSexp group into ARDSexp-survival and ARDSexp-dead groups according to different prognoses and performed PCA together with the control group (Figure 4A). The PCA results revealed that there was a relatively large taxonomic overlap between the control and ARDSexp-survival groups, whereas the ARDSexp-dead group had more distinct isolated specimens. This suggests that the control and ARDSexp-survival groups had a similar microbial community composition, whereas the ARDSexp-dead group had several abnormally increased background microorganisms.

Therefore, we compared the differences in the composition and abundance of microorganisms among the three groups. The Shannon index result indicated a similar diversity between the ARDSexp-survival, ARDSexp-dead, and control groups (Supplementary Figure 7C). The ARDSexp-dead group had significantly higher positive rates for *Escherichia coli* and *Haemophilus influenzae* than the control and ARDSexp-survival groups, respectively (Supplementary Figure 3A,B). However, no pathogenic microbial markers that might be associated with prognosis were identified.

**FIGURE 5:**
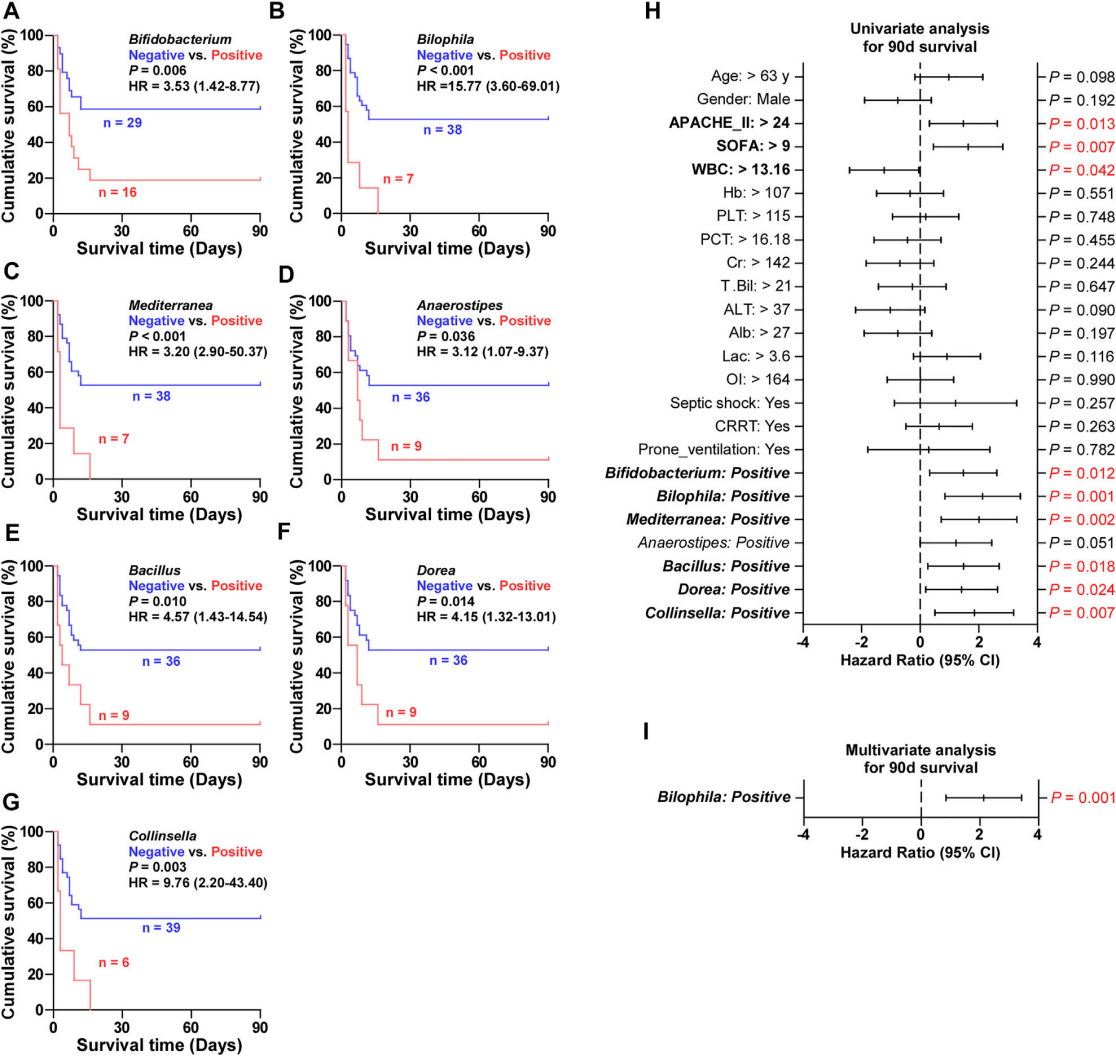
Validation of screened microbial markers associated with ARDSexp prognosis. **(A–G)** Kaplan–Meier analysis revealed that patients with one of the increased background microbiomes including *Bifidobacterium*, *Bilophila*, *Mediterranea*, *Anaerostipes*, *Bacillus*, *Dorea*, and *Collinsella*, had a shorter survival time. **(H)** Cox univariate analysis revealed that elevated pre-treatment APACHE II and SOFA scores and increased *Bifidobacterium*, *Bilophila*, *Mediterranea*, *Anaerostipes*, *Bacillus*, *Dorea*, and *Collinsella* were likely to be contributing factors to the death of ARDS patients. **(I)** Cox multivariate analysis revealed that *Bilophila* was the most significant and underlying risk factor for mortality in ARDS patients.

In addition, the ARDSexp-survival and ARDSexp-dead groups showed a predominant increase in the positivity and abundance of background microorganisms compared with the control group (Figures 4B–D and Supplementary Figure 3E,F). In contrast, the ARDSp group showed a decrease in these microorganisms. Notably, there was a simultaneous increase in the microorganisms of the ARDSexp-dead group including *Bifidobacterium*, *Bilophila*, *Mediterranea*, *Anaerostipes*, *Bacillus*, *Dorea*, and *Collinsella* (Figure 4E), when compared with the ARDSexp-survival and control groups. Further prognostic survival analysis was conducted and the results indicated that these seven microbes were strongly associated with poor prognosis (Figures 5A–G). In contrast, the Cox univariate analysis showed that increased pre-treatment APACHE II and SOFA scores and increased *Bifidobacterium*, *Bilophila*, *Mediterranea*, *Bacillus*, *Dorea*, and *Collinsella* may be risk factors for ARDS (Figure 5H). Furthermore, the Cox multivariate analysis also indicated that the increase in *Bilophila* was most likely associated with mortality in patients with ARDS (Figure 5I).

### 3.5 Changes in the Lung Microbiome Before and After Treatment in the ARDSp Group

To explore the changes in the lung microbiome before and after treatment in the ARDSp group, 30 cases with complete pre-and post-treatment comparative data were selected for analysis and screened for pathogenic organisms associated with death (potential risk factors for death) and markers associated with survival (potential protective factors for survival). Alpha diversity analysis showed that the ARDSp-preT group had lesser microbial diversity than the control group. There was a minor but not statistically significant increase in the microbial diversity after treatment compared with pre-treatment (Supplementary Figure 7D).

**FIGURE 6:**
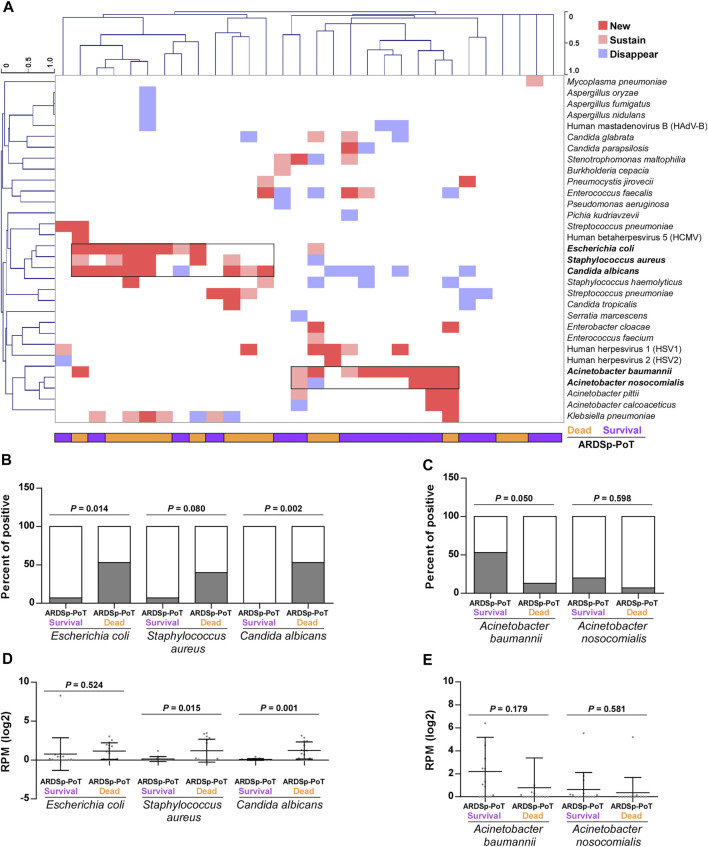
Changes in pulmonary pathogenic microorganisms before and after treatment in the ARDSp group (30 pairs). **(A)** Cluster analysis revealed that *Escherichia coli*, *Staphylococcus aureus,* and *Candida albicans* increased significantly in the ARDSp-poT-dead group, while *Acinetobacter baumanni*i and *Acinetobacter nosocomialis* increased or remained unchanged mainly in the ARDSp-poT-survival group. **(B–E)** Comparison of the positive rate and abundance of the aforementioned five pathogenic microorganisms in the ARDSp-poT-dead group versus the ARDSp-poT-survival group.

We conducted a cluster analysis to determine differences in pathogenic microorganisms and found that *Escherichia coli*, *Staphylococcus aureus*, and *Candida albicans* were significantly increased in the ARDSp-poT-dead group, whereas *Acinetobacter baumannii* and *Acinetobacter nosocomialis* were increased or unchanged, mainly in the ARDSp-poT-survival group (Figure 6A). An analysis of the microbial composition and abundance revealed increasing trends in positivity (*p* = 0.014, *p* = 0.080, and *p* = 0.002, respectively) and abundance (*p* = 0.524, *p* = 0.015, and *p* = 0.001, respectively) for *Escherichia coli*, *Staphylococcus aureus,* and *Candida albicans* in the ARDSp-poT-dead group (Figures 6B,D). The microorganisms that showed a significant increasing trend in positivity in the ARDSp-poT-dead group included *Klebsiella pneumoniae*, *Escherichia coli*, and *Candida albicans* (Supplementary Figure 4A). There was also a decreasing trend in the positivity and abundance of *Acinetobacter baumannii* and *Acinetobacter nosocomialis*; however, the result was not statistically significant (Figures 6C,E).

**FIGURE 7:**
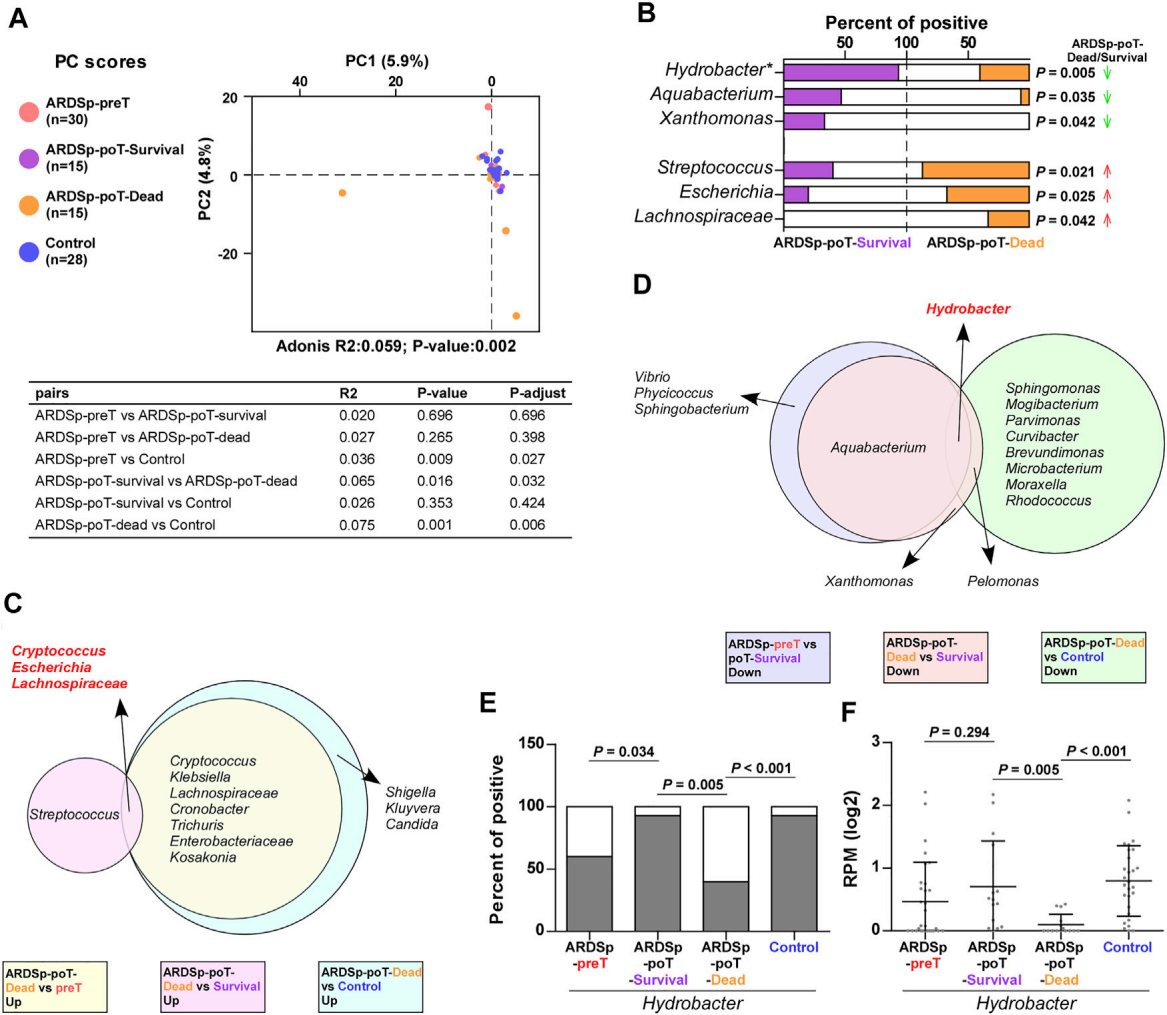
Changes in pulmonary background microorganisms before and after treatment in the ARDSp group (30 pairs). **(A)** PCA showed the community composition of the ARDSp-poT-dead group was different from that of the control group. **(B)** There were six background microorganisms with statistically significant positive rates in the ARDSp-poT-dead group compared with the ARDSp-poT-survival group. **(C)** Co-increased microorganisms in the ARDSp-poT-dead group compared with the ARDSp-preT group, the ARDSp-poT-dead group compared with the ARDSp-poT-survival group, and the ARDSp-poT-dead group compared with the control group included *Cryptococcus*, *Escherichia*, and Lachnospiraceae. **(D)** Commonly reduced microbiome in the ARDSp-preT group compared with the ARDSp-poT-survival group, the ARDSp-poT-dead group compared with the ARDSp-poT-survival group, and the ARDSp-poT-dead group compared with the control group were *Hydrobacter*. **(E,F)** Comparison of *Hydrobacter* in the four groups in terms of positive rate and abundance. R2: variation; P-adjust: *p* value was adjusted by using the Benjamini–Hochberg (BH) method.

In addition, the PCA showed that the ARDSp-poT-survival and control groups were relatively concentrated in background microorganism composition, and the ARDSp-poT-dead and ARDSp-preT groups had significantly distinct background microorganisms. These results indicated that the microbial community composition of the control and ARDSp-poT-survival groups were relatively similar, whereas the ARDSp-poT-dead and ARDSp-preT groups had several unusual background microorganisms (Figure 7A).

We speculated that the microorganisms that were increased in the ARDSp-poT-survival group compared with the ARDSp-preT group (Supplementary Figure 6E), especially those decreased in the ARDSp-poT-dead group compared with the ARDSp-poT-survival group and the control group (Figure 7B; Supplementary Figure 6C), were potential ARDSp protective factors. Notably, the most common microorganism was *Hydrobacter* (Figure 7D); *Hydrobacter* had the highest positivity or abundance values in the control group and decreased as prognosis deteriorated or increased as prognosis improved (Figures 7E,F). In contrast, we speculated that the microorganisms that were increased in the ARDSp-poT-dead group compared with the ARDSp-preT group (Supplementary Figure 6A), in the ARDSp-poT-dead group compared with the ARDSp-poT-survival group (Figure 7B), and an increase of in the ARDSp-poT-dead group compared with the control group (Supplementary Figure 6C) were potential risk factors for death in patients with ARDSp. The common microorganisms identified simultaneously were *Cryptococcus*, *Escherichia*, and Lachnospiraceae (Figure 7C), which showed the least difference in either positivity or abundance in the control group. These three microorganisms increased as the prognosis worsened or decreased as the prognosis improved (Supplementary Figure 4D-I).

## 4 Discussion

In this study, we investigated the changes in the lung microbiome of patients with sepsis-induced ARDS by mNGS sequencing. We compared the basic clinical characteristics and lung microbial composition of patients in each group and found significant differences between the ARDSp and ARDSexp groups. In addition, we found that the microbial diversity in ARDS induced by intrapulmonary infection was significantly decreased; however, there was no major difference among groups with different prognoses. The microbial profile of the ARDSp group was characterized by a reduced microbiome diversity, dominated by a decline in normal microbes in the lungs. The microbial profile of the ARDSexp group was characterized by an increased diversity of the microbiome, mainly in conditionally pathogenic bacteria and intestinal microbes. A comparison of the lung microbiome between the two groups indicated an increase in the pathogenic microorganisms *Escherichia coli*, *Staphylococcus aureus*, and *Candida albicans* in the lung, or an increase in enteric microbes or conditionally pathogenic bacteria, is potential a risk factor for death in ARDSp. The background microbiome *Hydrobacter* may be a protective factor for survival in ARDSp and the increase in *Bilophila* may be a mortality indicator in ARDSexp.

In addition, the differences in the microbiome between the ARDSp and ARDSexp groups at the baseline level were mainly manifested as differences in background bacterial microorganisms. The PCA results revealed significant differences in the microbial composition between the ARDSp, ARDSexp, and control groups. For example, the detection rate and abundance of the ARDSp microbiome were lower than those of the control and ARDSexp groups which had the highest detection rate and abundance, suggesting a decrease in the diversity of the ARDSp microbiome and an increase in the diversity of the ARDSexp microbiome. In ARDSexp, the epithelium and endothelium remain intact because of sepsis-induced increased vascular permeability and interstitial edema of the lungs; thus, the environment for the growth of the respiratory microbiome is not damaged. Increased vascular permeability leads to protein extravasation, which provides the necessary and abundant nutrients for bacterial growth to ensure that the normal respiratory microbiome is not reduced. The diversity of the microbiome possibly and significantly increases with the migration of the microbiome and the growth of environmental pathogens. Kyo et al., 2019analyzed the pulmonary microbiome of the BALF of patients with ARDS and discovered an increasing trend in pulmonary bacterial burdens, such as 16S rRNA gene copy number, and a pronounced decrease in α diversity. However, the results do not account for the origin of infection (pulmonary or extrapulmonary), and pneumonia was the main disease etiology, accounting for 65% of the cases. Dickson et al., 2016found that the lung microbiome was enriched with enteric bacteria and increased bacterial diversity in a mouse model of lung injury with abdominal sepsis caused by appendiceal ligation and puncture (Dickson et al., 2016), which is in accordance with our observations of the ARDSexp group. In our study, further analysis of the variation in the species of the ARDSp group versus the ARDSp group revealed that the background microorganisms *Hydrobacter*, *Sphingomonas*, *Curvibacter*, *Rhodococcus*, *Brevundimonas*, *Vibrio*, and *Microbacterium* were decreased in the ARDSp group compared with the control group. Several reports have indicated that *Sphingomonas*, *Brevundimonas*, and *Methylobacterium* are pulmonary microbes and conditionally pathogenic bacteria (Hilty et al., 2010; Huffnagle and Dickson, 2015; O'Dwyer et al., 2019). Therefore, the ARDSp microbiome is characterized by an increased abundance of pathogenic microorganisms and a decrease in other pulmonary respiratory microbiomes. In contrast, the ARDSexp group was characterized by an increase in different background microorganisms compared with the control group, both in terms of positivity and abundance. These microbes have been reported to be widespread, including oral and respiratory sources such as *Staphylococcus* and *Klebsiella* (Hostacka, 2001; Dyke, 2003) and intestinal microbes such as *Erwinia*, Lachnospiraceae, *Shigella*, and *Lactobacillus* (Starr and Chatterjee, 1972; Baker and The, 2018; Vacca et al., 2020). Hence, the microbiome of ARDSexp is characterized by an increase in the bacterial load, microbiome diversity, conditionally pathogenic bacteria, and intestinal microbes.

In addition, we compared the similarities and differences in the baseline levels of the lung microbiome in the survival and dead groups to screen for microbes associated with prognosis. In the ARDSp group, no microbe was identified. This result may be because the main etiology of ARDSp was severe pneumonia, which has a wide variety of infectious pathogenic microorganisms, and the infection characteristics and microbial features of different pathogenic microorganisms vary. Therefore, screening for meaningful microbes at the baseline is challenging. Future studies with a larger number of patients to quantity and stratify the analysis based on different pathogenic microorganisms, such as bacteria, fungi, and viruses are required to identify potential biomarkers. In the ARDSexp group, the microbiome was characterized by an increased lung bacterial load and microbiome diversity, and an increase in both conditionally pathogenic and enteric microbes and background microorganisms. Although pathogenic microorganisms related to prognosis were not identified, an increase in *Bilophila* among the background microbiome is most likely a risk factor for death in ARDSexp. *Bilophila*, a genus of intestinal microbes, can be isolated and cultured in abdominal infections, pulmonary infections, or infections at other sites. Moreover, the presence of increased bacterial load and Enterobacteriaceae in the pulmonary microbiome is associated with poor prognosis in patients with ARDS, which is consistent with our results. (Finegold et al., 1992; Cheung et al., 2019; Dahl et al., 2020). Therefore, an increase in *Bilophila* is a potential indicator for ARDSexp mortality.

ARDSp cases with complete data before and after treatment were selected for analysis and comparison. A cluster analysis of pathogenic bacteria showed that *Escherichia coli*, *Staphylococcus aureus,* and *Candida albicans* were more abundant in the ARDSp-poT-dead group than in the ARDSp-preT group. The ARDSp-poT-dead group exhibited increased positivity or abundance of *Escherichia coli*, *Candida albicans*, *Staphylococcus aureus*, and *Klebsiella pneumoniae* compared with the ARDSp-poT-survival group. These results suggest that the pathogenic bacteria *Escherichia coli*, *Staphylococcus aureus*, and *Candida albicans* are potential risk factors for ARDSp death. In a retrospective analysis of the anti-infective regimens for these patients, we found that all regiments included treatment for *Escherichia coli* and *Staphylococcus aureus* and that 53.3% of non-surviving patients had received treatment for *Candida albicans*. These findings suggest that the increase in these pathogens was not strongly correlated with the anti-infective regimen, indicating a poor prognosis of ARDSp. *Escherichia*, Lachnospiraceae, *Cryptococcus,* and other enteric microbes and conditionally pathogenic bacteria may be associated with the risk of death in ARDSp, which is consistent with previous studies showing that the lung microbiome was enriched in enteric bacteria in mouse models of sepsis and patients with ARDS. Intestinal-specific microbes (*Bacteroides*) are common and abundant in the BALF of patients with ARDS and correlate with the intensity of systemic inflammation (Dickson et al., 2016), suggesting an interaction between the lower respiratory tract and the gastrointestinal tract. Dickson et al., 2020reported that increased bacterial load and gut-associated bacterial enrichment help predict the prognosis of patients with ARDS. In addition, the presence of *Candida* in septum cultures is associated with increased mortality in immunosuppressed patients (Pendleton et al., 2018). This observation may support the hypothesis that the increase in the pathogenic bacteria *Escherichia coli*, *Staphylococcus aureus,* and *Candida albicans*, and the increase in the intestinal microbiome, are important contributors to mortality in ARDSp; therefore, treatment targeting the intestinal microbiome in patients with ARDS should be considered.

We identified *Hydrobacter* as a possible protective factor against ARDSp. *Hydrobacter* was the most abundant in the control group and was associated with a better prognosis (Figures 7E,F). Little attention has been paid to the role of microbes such as *Hydrobacter* in the lower respiratory tract; however, the presence of a “normal microbiome” in the respiratory tract may be closely associated with the pathogenesis and development of ARDSp. *Hydrobacter* can be found in pure water (Eder et al., 2015); however, it remains unknown whether it belongs to the normal microbiota of the respiratory tract and whether it acts alone or in concert with other microbiomes. Extensive studies have demonstrated the positive effects of commensal microorganisms on human health (Nembrini et al., 2011; Fyhrquist et al., 2014; Zhang et al., 2021); therefore, microbial agents have potential clinical applications in maintaining lung function. Nevertheless, further studies are required to determine whether microbes such as *Hydrobacter* can serve as therapeutic targets for ARDS.

This study was limited by the absence of RNA sequencing data, missing information on RNA viruses, and microbial transcriptome alterations. The lack of 16s rRNA sequencing for microbial analyses hinders comparisons of the total bacterial abundance (using quantitative PCR), relative abundance (taxonomic composition of the specimen community), and community characteristics (e.g., diversity) for the whole sample. Greater efforts are needed to combine 16S rRNA and metagenomic sequencing to conduct more precise analyses of the community composition, diversity, evolutionary relationships, and gene functions. In addition, the results were potentially biased owing to inadequate sample quantity, the influence of retrospective analysis, and numerous clinical factors. Moreover, the application of BALF must consider the risk of sample contamination. However, in this study, all patients received an aseptically operated endotracheal tube; therefore, the risk of bacterial contamination was considered minimal. Antibiotic treatments may also affect alterations in the respiratory microbiome; however, previous studies have shown that in patients with traumatic ARDS, the antibiotic application is not significantly linked to the composition of the respiratory microbiome (Panzer et al., 2018). In addition, the patients were treated according to the criteria of the sepsis guidelines; thus, we believe that the impact of antibiotics was minimal. Further multicenter and prospective controlled studies are needed to recruit more patients and subdivide infections at different sites and with different pathogens to better understand the microbial profile of ARDS with different etiologies.

In general, the interaction or synergy between the lung microbiome and gut microbiome plays a regulatory role in the inflammatory immune response of the body. The association between microorganisms and their hosts is intricate and poorly understood. Their interaction should be balanced and mutually constrained, implying that no single microbe can affect the microbial function completely, while changes in any one part can influence the development of health and disease. To date, it remains unclear whether alterations in the microbial community in one region affect other regions or whether systemic effects produce a specific microbial community in a specific tissue (Lyon, 2017). Moreover, no systematic or large-scale studies have been undertaken; therefore, further longitudinal studies should be performed to correlate microorganisms with the severity of lung disease in humans and animals, thereby facilitating their application in etiological determination and disease control.

## Data Availability

The clinical information presented in the study is included in the article/Supplementary Material, further inquiries can be directed to the corresponding authors.
