# Supplementary figures and images for: Pulmonary Microbial Composition in Sepsis-Induced Acute Respiratory Distress Syndrome
Source: Front Mol Biosci. 2022 Jun 23;9:862570. doi: 10.3389/fmolb.2022.862570 (PMC9262094; doi:10.3389/fmolb.2022.862570)

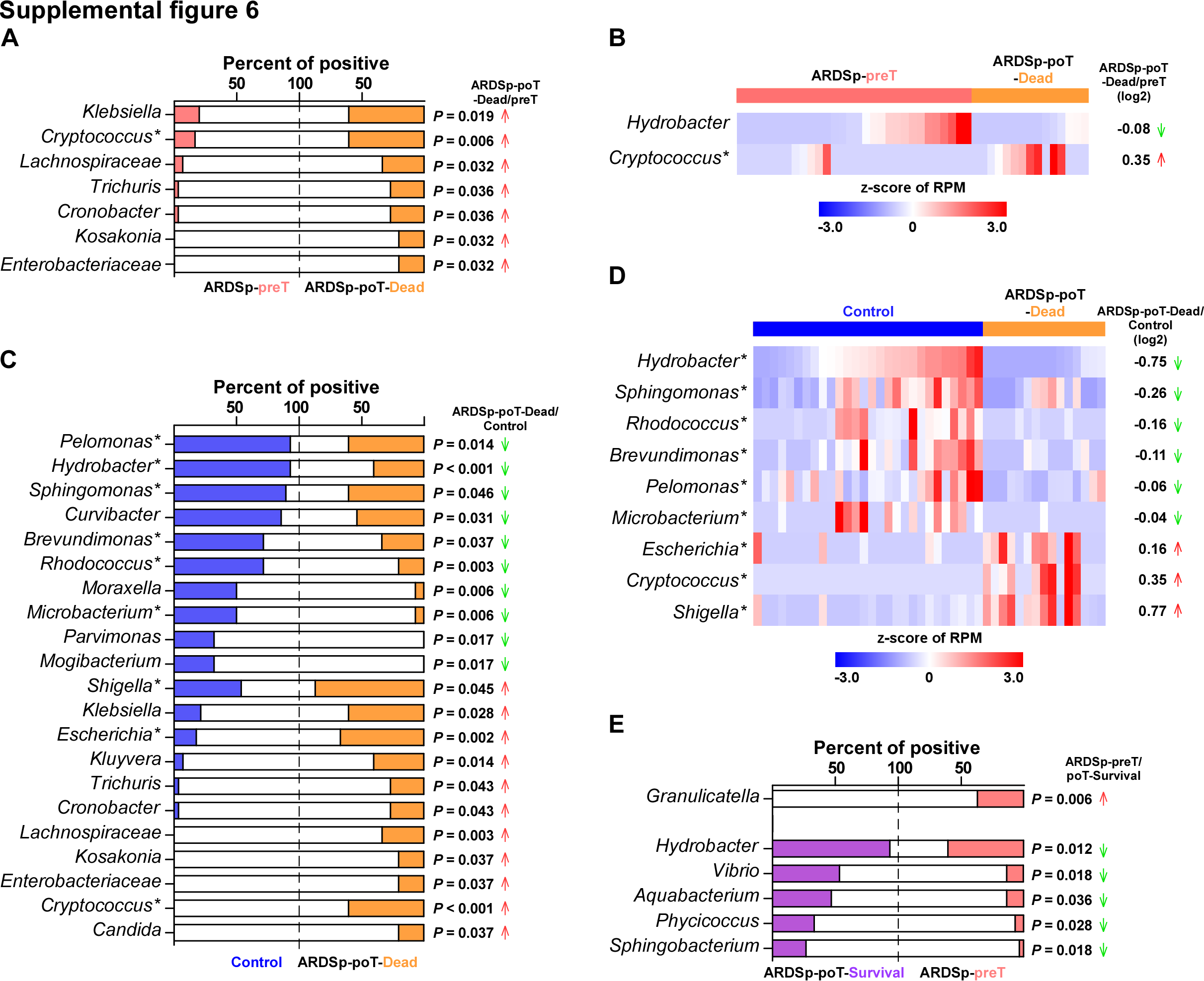

Supplement: Supplementary file 1 [file Image6.TIF]

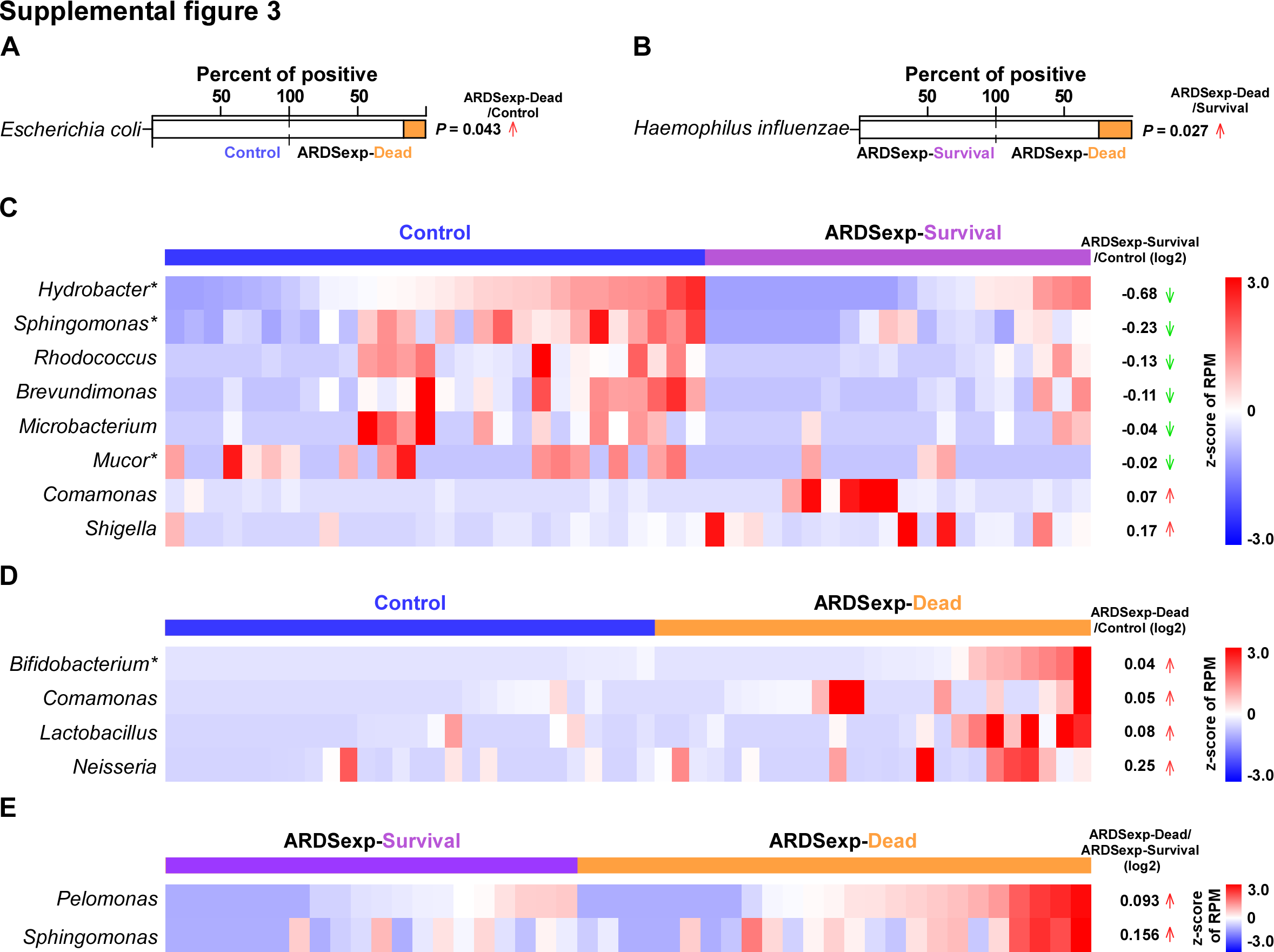

Supplement: Supplementary file 3 [file Image3.TIF]

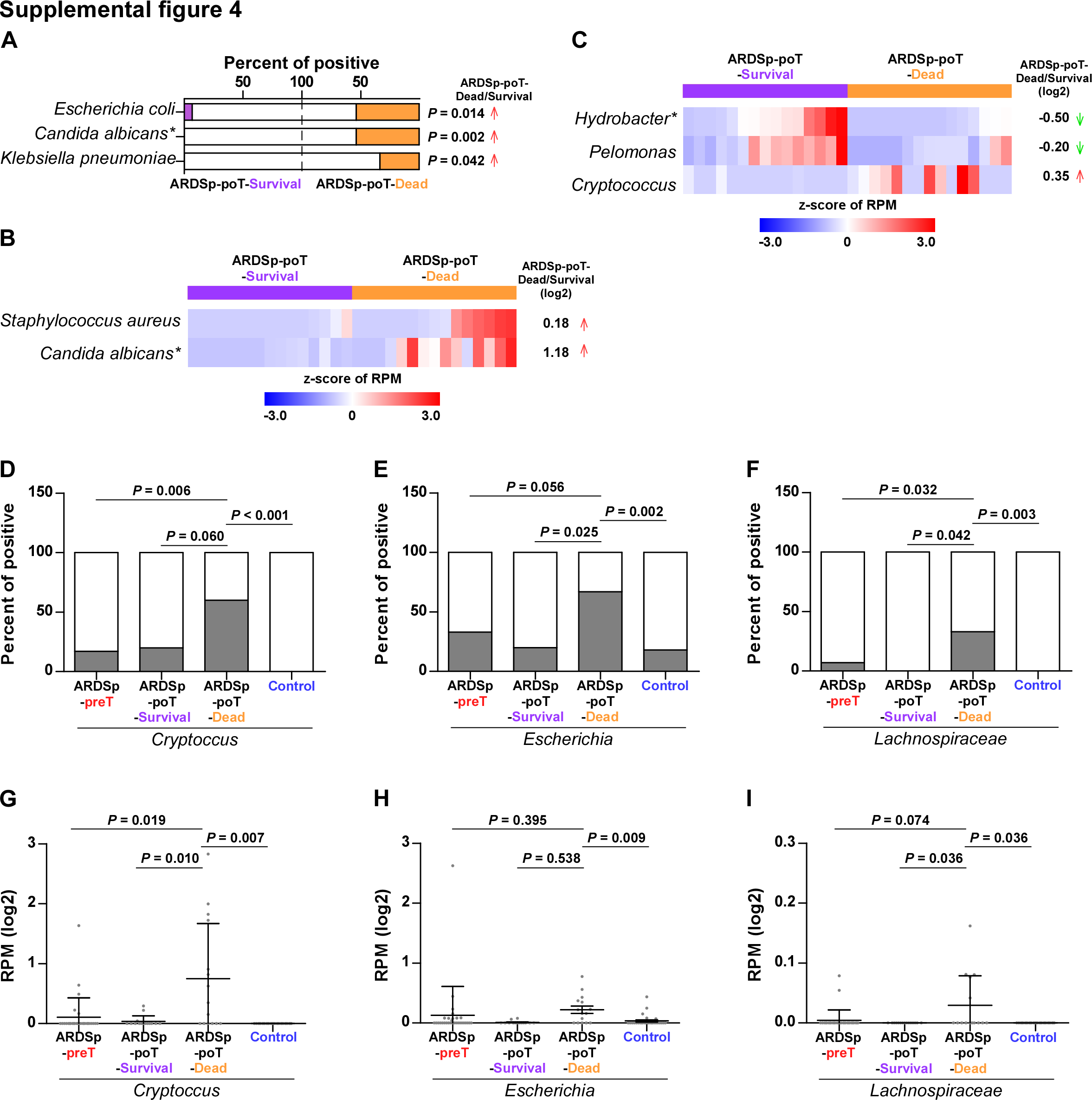

Supplement: Supplementary file 4 [file Image4.TIF]

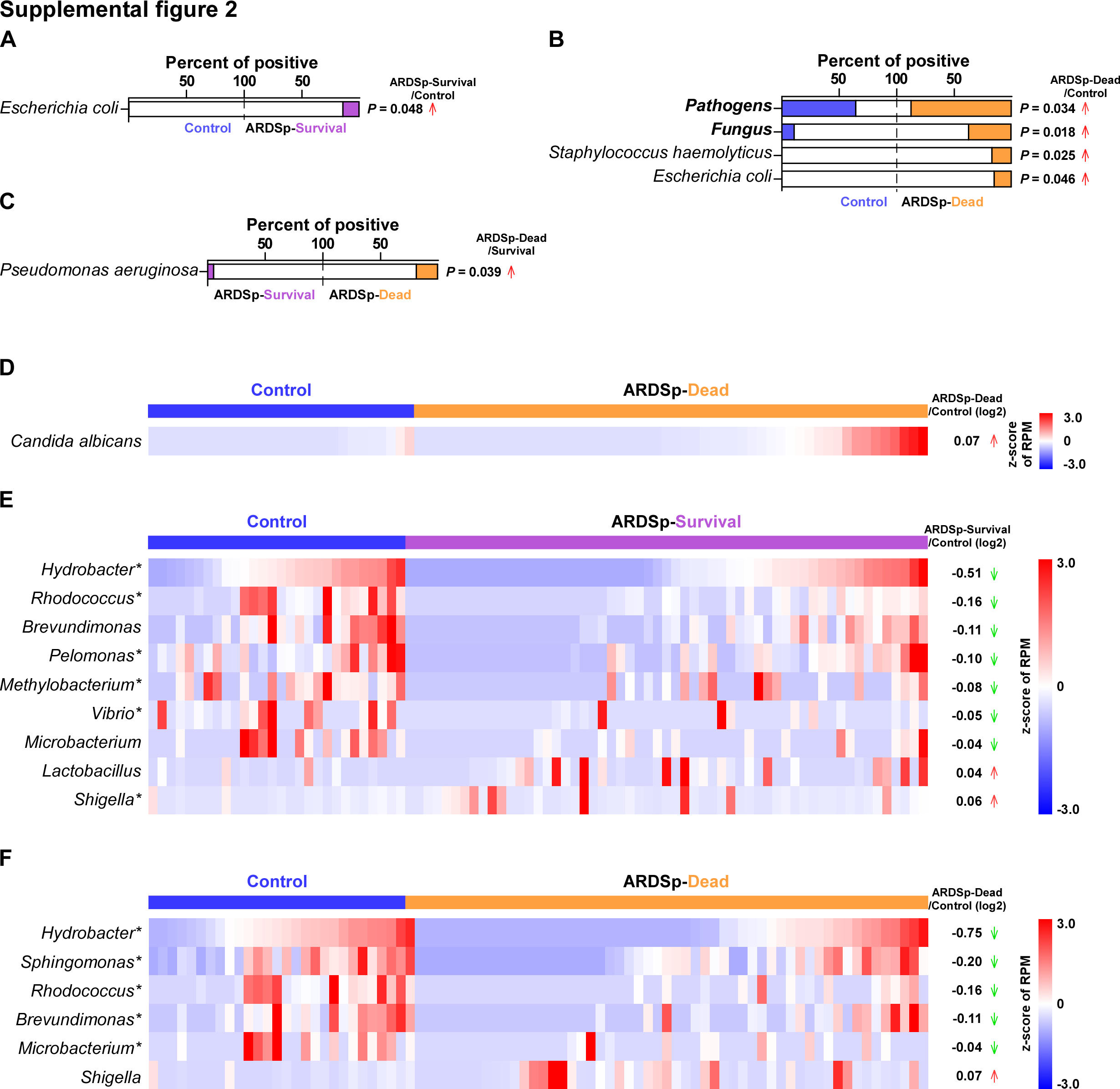

Supplement: Supplementary file 5 [file Image2.TIF]

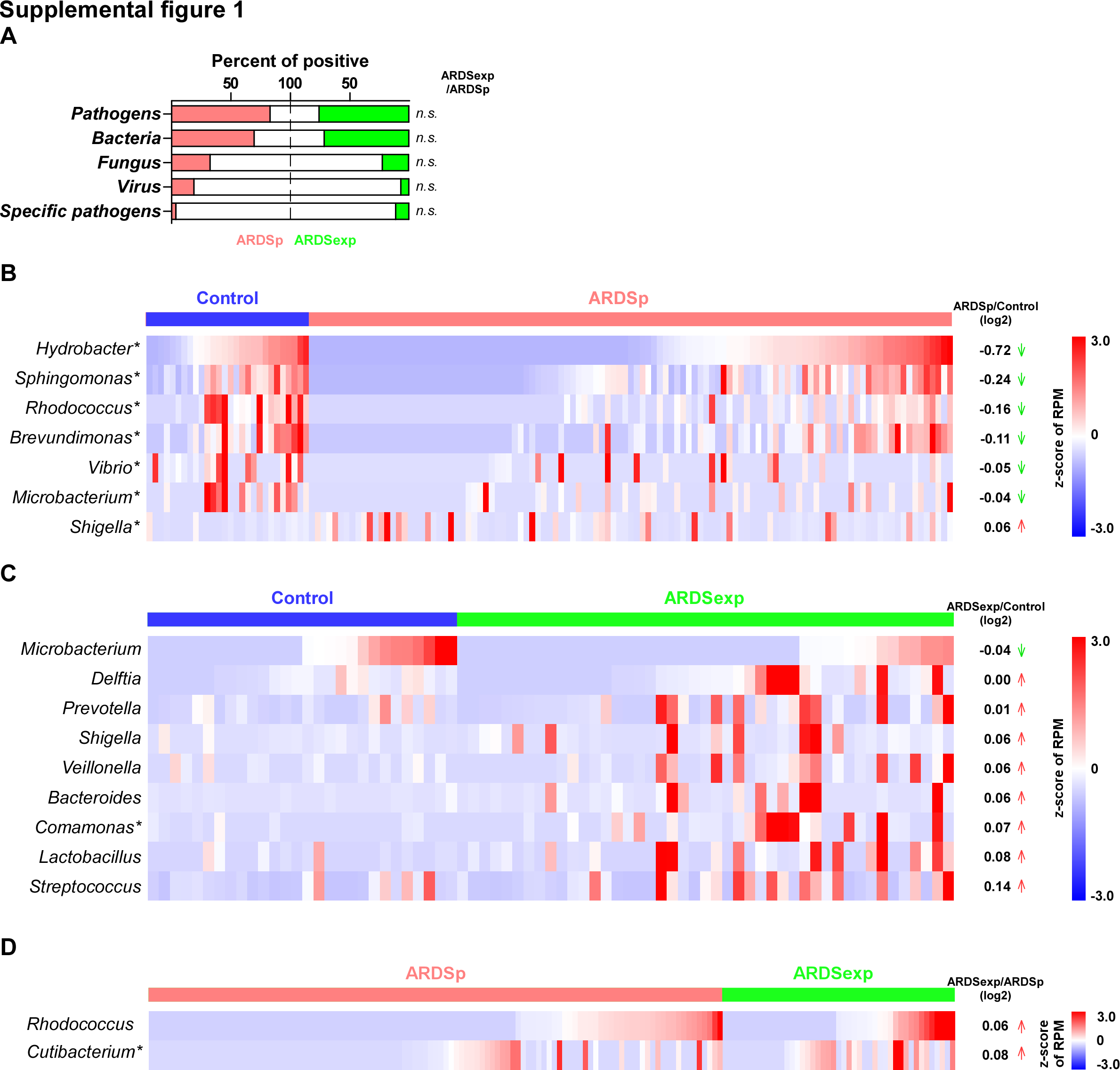

Supplement: Supplementary file 6 [file Image1.TIF]

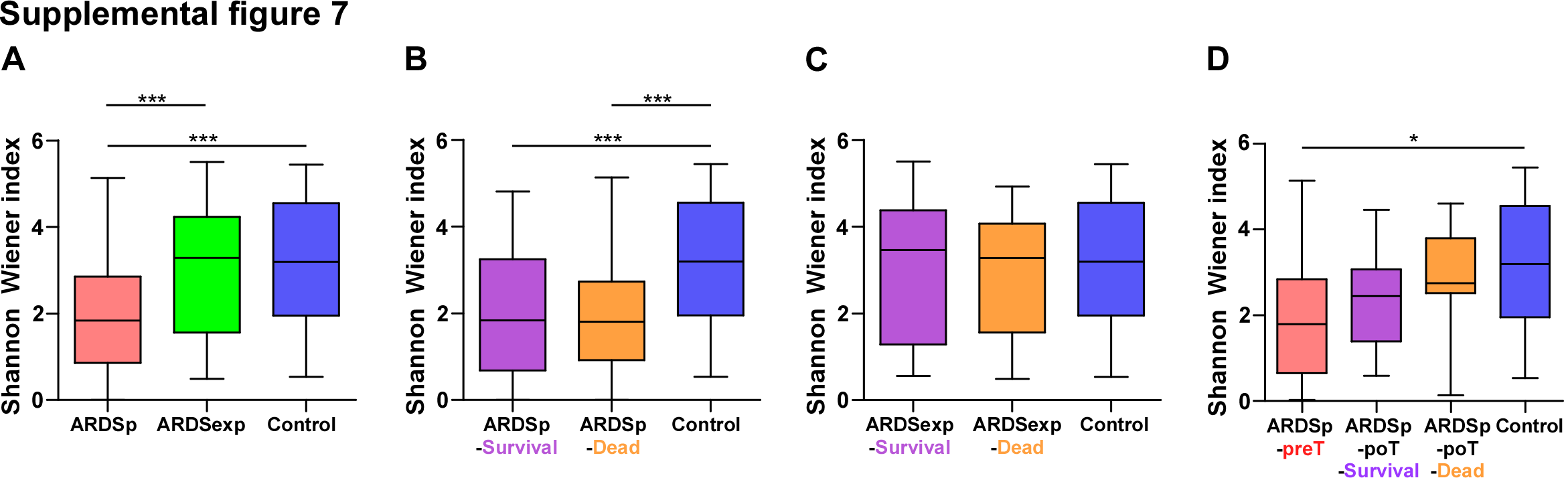

Supplement: Supplementary file 7 [file Image7.TIF]

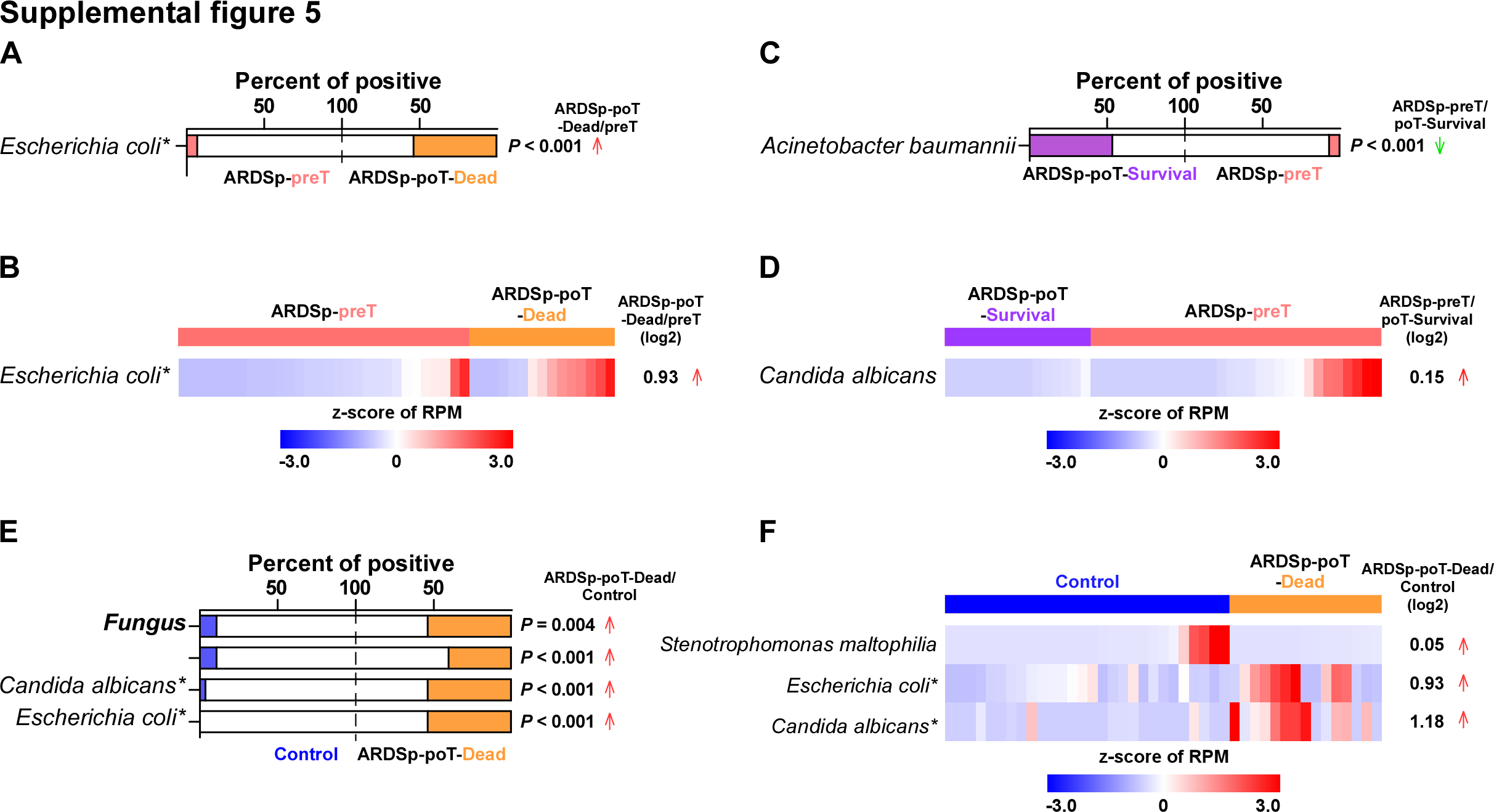

Supplement: Supplementary file 8 [file Image5.TIF]
