# Supplementary material for: Pulmonary Microbial Composition in Sepsis-Induced Acute Respiratory Distress Syndrome
Source: Front Mol Biosci. 2022 Jun 23;9:862570. doi: 10.3389/fmolb.2022.862570 (PMC9262094; doi:10.3389/fmolb.2022.862570)
Supplement: Supplementary file 2 [file Table1.docx]

**Supplemental table 1 Common pathogenic microorganisms.**

| **[Bacteria](javascript:;)** | | **[Fungus](javascript:;)** | **[Virus](javascript:;)** | **Specific pathogen** |
| --- | --- | --- | --- | --- |
| **Gram-positive bacteria** | **Gram-negative bacteria** |  |  |  |
| *Streptococcus pneumoniae* | *Escherichia coli* | *Candida albicans* | *Human herpesvirus 1 (HSV1)* | *Mycobacterium tuberculosis* |
| *Streptococcus pyogenes* | *Acinetobacter baumannii* | *Candida glabrata* | *Human herpesvirus 2 (HSV2)* | *Mycobacterium abscessus* |
| *Staphylococcus epidermidis* | *Acinetobacter calcoaceticus* | *Candida parapsilosis* | *Human herpesvirus 4 (EBV)* | *Mycobacterium intracellulare* |
| *Staphylococcus aureus* | *Acinetobacter pittii* | *Candida orthopsilosis* | *Human herpesvirus 5 (CMV)* | *Mycobacterium kansasii* |
| *Staphylococcus hominis* | *Acinetobacter nosocomialis* | *Candida tropicalis* | *Human herpesvirus 6B* | *Mycoplasma orale* |
| *Staphylococcus haemolyticus* | *Pseudomonas aeruginosa* | *Aspergillus flavus* | *Human herpesvirus 7* | *Mycoplasma pneumoniae* |
| *Staphylococcus capitis* | *Klebsiella pneumoniae* | *Aspergillus fumigatus* | *Human mastadenovirus C* | *Chlamydia pneumoniae* |
| *Staphylococcus intermedius* | *Haemophilus influenzae* | *Aspergillus nidulans* | *Human mastadenovirus B* | *Orientia tsutsugamushi* |
| *Enterococcus faecalis* | *Enterobacter cloacae* | *Aspergillus terreus* | *Human mastadenovirus E* |  |
| *Enterococcus faecium* | *Proteus mirabilis* | *Aspergillus oryzae* |  |  |
|  | *Stenotrophomonas maltophilia* | *Aspergillus niger* |  |  |
|  | *Burkholderia cepacia* | *Saccharomyces cerevisiae* |  |  |
|  | *Serratia marcescens* | *Pichia kudriavzevii* |  |  |
|  | *Legionella pneumophila* | *Cryptococcus neoformans* |  |  |
|  |  | *Pneumocystis jirovecii* |  |  |
|  |  | *Scedosporium apiospermum* |  |  |

**Supplemental table 2 Primers for PCR.**

| **Primer** | **Sequence** |
| --- | --- |
| M16S-F | TCGTCGGCAGCGTCAGATGTGTATAAGAGACAGCCTACGGGNGGCWGCAG |
| M16S-R | GTCTCGTGGGCTCGGAGATGTGTATAAGAGACAGGACTACHVGGGTATCTAATCC |

PCR: polymerase chain reaction
